# IL-5 drives eosinophils from bone marrow to blood and tissues in a guinea-pig model of visceral larva migrans syndrome

**DOI:** 10.1155/S096293519600004X

**Published:** 1996-02

**Authors:** L. H. Faccioli, V. F. Mokwa, C. L. Silva, G. M. Rocha, J. I. Araujo, M. A. Nahori, B. B. Vargaftig

**Affiliations:** 1 Department of Parasitology Microbiology and Immunology School of Medicine of Ribeirão Preto Ribeirão Preto SP 14049-900 Brazil; 2 Unité de Pharmacologie Cellulaire Unité Associée Institut Pasteur/INSERM n. 285 Paris France

## Abstract

This study was undertaken to evaluate the role of IL-5 in eosinophil migration and in the maintenance of eosinophilia in a guinea-pig model of visceral larva migrans syndrome. The results show that the infection of animals with *Toxocara canis* induced an early increase in serum IL-5 levels that might be essential for eosinophil differentiation and proliferation and for the development of eosinophilia. When infected guinea-pigs were treated with mAb anti-IL-5 (TRFK-5) given at the same time or 1 or 3 days after infection, there was a high percentage of reduction of eosinophil counts 18 days after infection. However, when the mAb was administered during the peak of eosinophilia, there was high inhibition in blood, no inhibition in bronchoalveolar lavage fluid (BALF) or peritoneum and an increase in eosinophil numbers in bone marrow. Thus, a basic level of IL-5 may be essential to drive eosinophils from bone marrow to blood and tissues, and for the maintenance of eosinophilia in infected animals. We may also conclude that when eosinophils have already migrated to the lungs, TRFK-5 has no power to inhibit eosinophilia, which is also under control of local lung cells producing IL-5. In this way, only one later TRFK-5 treatment may not be sufficient to modify the lung parenchyma microenvironment, since *T. canis* antigens had already stimulated some cell populations to produce IL-5.


Mediators of Inflammation 5, 24–31 (1996)

### IL-5 drives eosinophils from bone marrow to blood and tissues in a guinea-pig model of visceral larva migrans syndrome

L. H. Faccioli,[1,CA] V. F. Mokwa,[1] C. L. Silva,[1] G. M. Rocha,[1] J. I. Araujo,[1] M. A. Nahori[2] and B. B. Vargaftig[2]

[1]Department of Parasitology, Microbiology and Immunology, School of Medicine of Ribeirão Preto, 14049-900, Ribeirão Preto, SP, Brazil. Fax: (+55) 16 633 6631 ; [2]Unité de Pharmacologie Cellulaire, Unité Associée Institut Pasteur/INSERM n. 285, Paris, France.

[CA]Corresponding Author

**This study was undertaken to evaluate the role of IL-5 in eosinophil migration and in the maintenance of eosinophilia in a guinea-pig model of visceral larva migrans syndrome. The results show that the infection of animals with *Toxocara canis* induced an early increase in serum IL-5 levels that might be essential for eosinophil differentiation and proliferation and for the development of eosinophilia. When infected guinea-pigs were treated with mAb anti-IL-5 (TRFK-5) given at the same time or 1 or 3 days after infection, there was a high percentage of reduction of eosinophil counts 18 days after infection. However, when the mAb was administered during the peak of eosinophilia, there was high inhibition in blood, no inhibition in bronchoalveolar lavage fluid (BALF) or peritoneum and an increase in eosinophil numbers in bone marrow. Thus, a basic level of IL-5 may be essential to drive eosinophils from bone marrow to blood and tissues, and for the maintenance of eosinophilia in infected animals. We may also conclude that when eosinophils have already migrated to the lungs, TRFK-5 has no power to inhibit eosinophilia, which is also under control of local lung cells producing IL-5. In this way, only one later TRFK-5 treatment may not be sufficient to modify the lung parenchyma microenvironment, since *T. canis* antigens had already stimulated some cell populations to produce IL-5.**

**Key words:** Eosinophil, Eosinophilia by *Toxocara canis*, IL-5 in eosinophilia, *Toxocara canis*

### Introduction

Eosinophilia has been associated with parasitic diseases, particularly when the parasites invade the tissues or injure the mucosal surfaces.[1] *Toxocara canis* is an intestinal parasite of dogs, and is the most common aetiologic agent of visceral larva migrans syndrome (VLMS). In humans, VLMS results from the ingestion of embryonated eggs of *T. canis*, that eclode in the small intestine. The infective larvae invade the mucosa, move into the liver via the portal circulation, and from there to the lungs.[2] Beaver *et al.*,[3] who were the first to describe this syndrome, noted the intense eosinophilia which reaches more than 90% of total leucocyte counts. However, there are few studies regarding the mechanisms involved in the blood and tissue eosinophilia observed in VLMS.

Several investigators have suggested a direct correlation between eosinophilia and interleukin-5 (IL-5) in human helminth infections[4,5] and in experimental animal models.[6,7] Inhibition of eosinophilia has been demonstrated by anti-IL-5 treatment in mice infected with *Nippostrongylus brasiliensis*,[8] *Schistosoma mansoni*,[9] *Toxocara canis*[7] and *Heligmosomoides polygyrus*.[10] IL-5 has also been shown to support the terminal differentiation, proliferation of eosinophil precursors[11,12] and eosinophil activation.[13] Although IL-5 does not demonstrate eosinophil chemotactic activity *in vivo*[14] there is some evidence suggesting that this cytokine may modulate a selective eosinophil accumulation at the site of inflammation. Moreover, Sehmi *et al.*[15] reported that IL-5 has a selective priming effect on eosinophil migratory response to nonselective chemoattractant mediators *in vitro*. Also, Moser *et al.*[16] have demonstrated that in order to acquire the ability to transmigrate, eosinophils must be primed with IL-5, IL-3 and GM–CSF. Thus, the involvement of IL-5 in eosinophilia is not fully understood.

In the present study we have used a guinea-pig model of VLMS to investigate the involvement of IL-5 in eosinophil migration and in the maintenance of eosinophilia in blood, bone marrow, lung and peritoneal cavity.

© 1996 Rapid Science Publishers

### Materials and Methods

*Animals:* Outbred albino female guinea-pigs weighing 300–400 g at the start of the experiments were obtained from the animal house of the School of Medicine of Ribeirão Preto, University of São Paulo, Brazil.

*Infection of animals: T. canis* eggs were obtained by the methods of Olson and Schulz,[17] with minor modifications. Briefly, gravid female worms were recovered from dogs, and the eggs were rescued from the uterus, washed and allowed to develop to the infective stage in shallow dishes containing 0.5% formalin at 37°C. Under light ether anaesthesia, the animals were infected with 1 ml saline containing 500 *T. canis* eggs, by gastric intubation using a metal cannula.

*Blood cell counts:* Guinea-pigs were anaesthetized with sodium pentobarbitone (30 mg/kg, i.v.) and blood samples were collected by cardiac puncture with 10% EDTA. Total cell counts were carried out using diluting fluid in a Neubauer chamber. Differential countings were obtained using Rosenfeld-stained cytocentrifuge preparations.

*Bronchoalveolar lavage fluid:* The guinea-pigs were killed by an overdose of sodium pentobarbitone and 5 ml of phosphate-buffered saline (PBS) containing 0.5% sodium citrate (PBS/SC), at room temperature, were instilled through a polyethylene cannula introduced into the trachea. The cells present in the bronchoalveolar lavage fluid (BALF) were recovered immediately. The procedure was repeated once. The leucocyte counts in the BALF were determined as described above.

*Peritoneal cells:* The cells from the peritoneal cavities were harvested by injection of 10 ml of PBS/SC into the peritoneum. Only 5–8 ml of the fluid was withdrawn for cell counts, as described above.

*Bone marrow cells:* Bone marrow cells were collected by flushing the contents of the guinea-pig femur with 10 ml of PBS/SC. Total cell numbers were determined as above. In the differential cell counts the cell populations were divided into mature neutrophils, mature eosinophils and others (mainly precursors and mononuclear cells).

*Histopathological studies:* Tissues were removed from guinea-pigs at various times post-infection and immediately fixed in 10% formalin. Tissues were routinely processed, embedded in paraffin, sectioned at 4–6 µm, and stained with Chromothope 2R and haematoxylin, for examination by light microscopy.

*Determination of IL-5 in serum:* The IL-5 level in the serum of guinea-pigs was measured using an enzyme-linked immunosorbent assay (ELISA). Briefly, ELISA plates (96-well Immunoplate Maxi-Sorp, Nunc, Roskilde, Denmark) were coated with IL-5-specific monoclonal antibody (TRFK-5, 5 µg/ml in phosphate buffered saline, pH 7.4, PBS, 100 µl/well). After 2 h of incubation at 37°C, the wells were washed four times with PBS containing 0.1% Tween 20 (PBS-T). Then, 100 µl of samples or recombinant murine IL-5 standards (0.15–200 ng/ml) in PBS-T and 2% BSA (PBS–TBSA) were added to each well. After incubation for 1 h at 37°C, the wells were washed three times and 100 µl of biotinylated rat anti-murine IL-5 (biotinylated-TRFK-5, 1 µg/ml in PBS–TBSA) was added. After incubation for 1 h at 37°C, the wells were washed again three times and 100 µl of peroxidase-labelled streptavidin (1/1000, Kirkegaard & Perry Laboratories Inc., Maryland, USA) were added to each well. Following incubation for 1 h at 37°C and further washing, the enzyme was developed using the TMB substrate peroxidase for 5 min. The reaction was stopped by adding 50 µl of 2.0 N HCl, and the optical densities were read at 490 nm using an automated plate reader. The sensitivity of the assay was 0.15 ng/ml and the upper limit 100 ng/ml.

*Monoclonal antibodies:* The rat monoclonal antibody TRFK-5 was a generous gift from Dr P. Minoprio, Institut Pasteur, Paris. The neutralizing antibody was purified by precipitation with ammonium sulfate (45%) from ascites prepared in CD1 nude mice (Charles River, St Aubin les Elbeuf, France) inoculated 1 week before the injection of hybridoma cells, with 1 ml of pristane (Sigma). After precipitation and dialysis of the ascite fluid overnight against PBS, the dialysate was further purified on a Protein G1 column (HiTrap™, Pharmacia Upsala, Sweden).

*Eosinophil and cytokine depletion:* Guinea-pigs were injected i.p. with TRFK-5 or with the irrelevant antibody (rat IgG against total anti-human IgG) once, 2 mg/animal, at the time of infection or at different intervals (1, 3, 12 or 17 days) thereafter. The animals in this group were sacrificed 18 days after infection.

*Recovery of larvae from liver:* One lobule of each liver was used to determine the larval counts from infected guinea-pigs. Larval recovery was evaluated as described by Kayes and Oaks,[18] with minor modifications. Briefly, the tissue was chopped and digested with pepsin–HCl (pH 1.5–1.8) for 2 h at 37°C. Larval counts for each sample were performed after centrifugation and examination of three 100-μl samples under the light microscope.

*Statistical analysis:* Data are presented as the mean ± S.E.M. and were analysed statistically using the Mann–Whitney test for unpaired data. A $p < 0.05$ value was considered to be statistically significant.

### Results

*Kinetics of eosinophil counts in blood, bone marrow, BALF and peritoneum:* Guinea-pigs infected with *T. canis* eggs showed a time-dependent blood, bone-marrow, BALF and peritoneal eosinophilia (Fig. 1). The results represent the mean of nine animals obtained in three different experiments. The eosinophil number increased significantly from $0.55 \pm 0.37 \times 10^5$ at the beginning of experiment to $6.0 \pm 1.03 \times 10^5$ at 6 days post-infection, peaked at day 18 ($12.0 \pm 2.31 \times 10^5$), and decreased by day 24 ($8.11 \pm 2.85 \times 10^5$) (Fig. 1A). A rise in the percentage of mature eosinophils in bone marrow was observed 12 days after infection (ranging from 6 ± 2% to 14 ± 2%) and peaked at 18 days (17 ± 2%) (Fig. 1B). As in blood, the number of eosinophils in BALF increased significantly from $0.14 \pm 0.06 \times 10^5$ to $1.37 \pm 0.35 \times 10^5$ at 6 days after infection, reaching a peak at 18 days ($10.23 \pm 2.62 \times 10^5$) with an increase in relative number of as much as 90% in eosinophil counts in relation to controls, and was still elevated at day 24 ($9.07 \pm 3.47 \times 10^5$) (Fig. 1C). The remaining cells in the BALF were alveolar macrophages, lymphocytes, mast cells and ciliated cells. In contrast to blood and BALF, the number of eosinophils in the peritoneal cavity increased significantly only at day 12 post-infection (onset, $2.06 \pm 1.04 \times 10^5$; day 6, $3.68 \pm 0.82 \times 10^5$; day 12, $5.77 \pm 1.12 \times 10^5$; and increased progres-

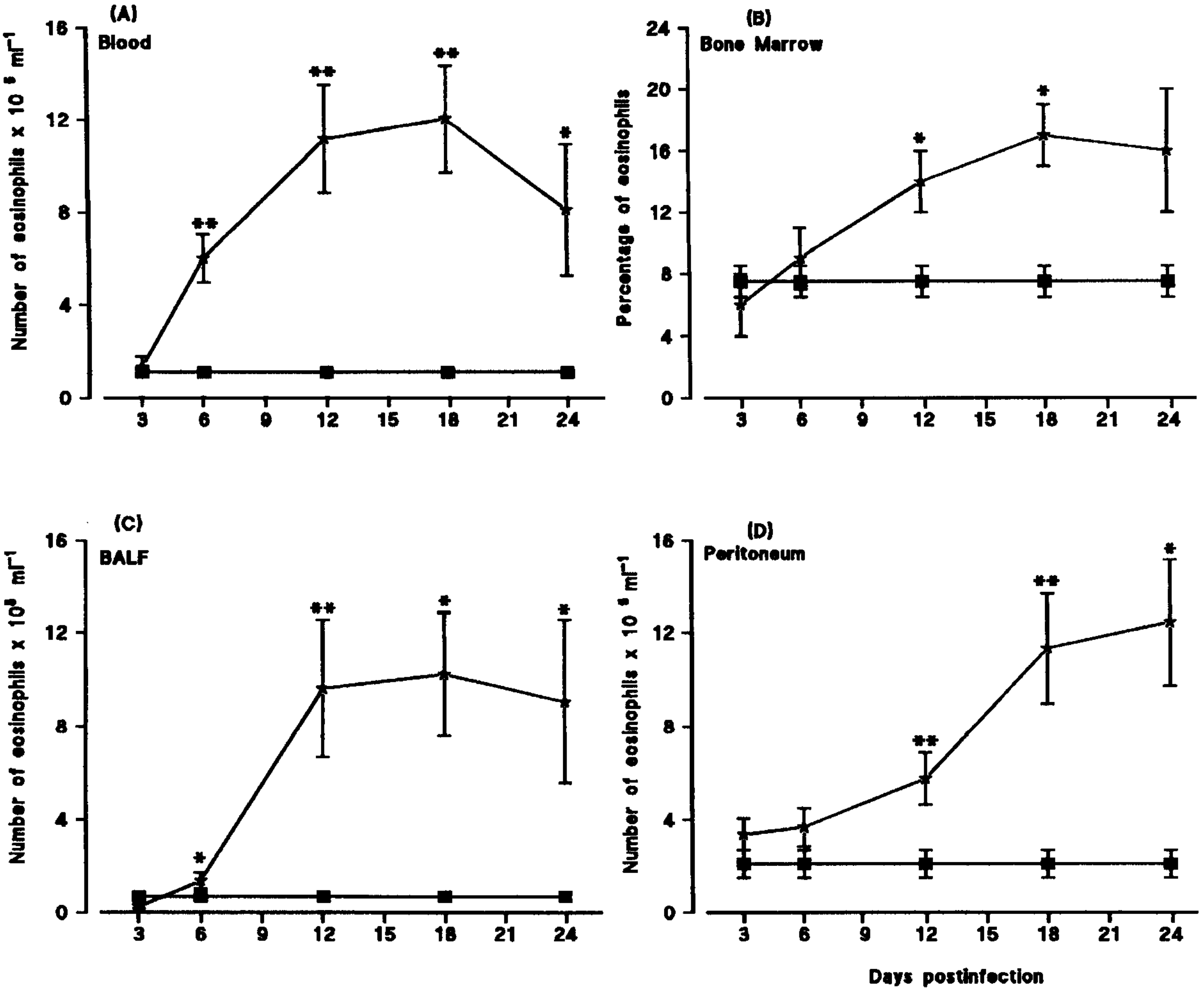

FIG. 1. Number of eosinophils in blood, BALF and peritoneal cavity, and percentage of eosinophils in bone marrow of *T. canis*-infected guinea-pigs. Values are the mean ± S.E.M. ($n = 8$ to 9). Asterisks indicate a significant difference between infected and noninfected animals ($n = 5$–6). *$p < 0.05$ and **$p < 0.01$.

sively until day 24, 12.44 ± 2.72 × $10^5$) (Fig. 1D). The percentage of eosinophils in some animals reached 55% at the peak of infection. No increase in the number of mononuclear cells was seen in any compartment analysed.

*Larval counts:* The percentage of inoculated *T. canis* larvae recovered by peptic digestion of the liver of experimental animals 4 h and 1, 2, 3, 4, 9, 12 and 18 days after inoculation of 500 eggs per animal is shown in Fig. 2. Most of the larvae were recovered 2 to 4 days after infection and 10% recovery was also observed on day 18 in the liver of the animals.

*IL-5 level in serum of infected animals:* IL-5 was measured in the serum of infected and normal guinea-pigs. Each time point in Fig. 3 represents the mean of results from three to five infected animals, and from six controls. Two peaks of IL-5 were present in the serum of infected guinea-pigs 1 day after infection (102 ± 22 pg/ml), and 18 days later (59 ± 7 pg/ml). The level of IL-5 in the controls was 31 ± 4 pg/ml.

*Eosinophil numbers in infected animals treated with TRFK-5:* When guinea-pigs received an i.p. injection of TRFK-5, the monoclonal antibody against IL-5, at the time of egg administration or 1 day later, the number of eosinophils in blood, BALF, peritoneal cavity and bone marrow was

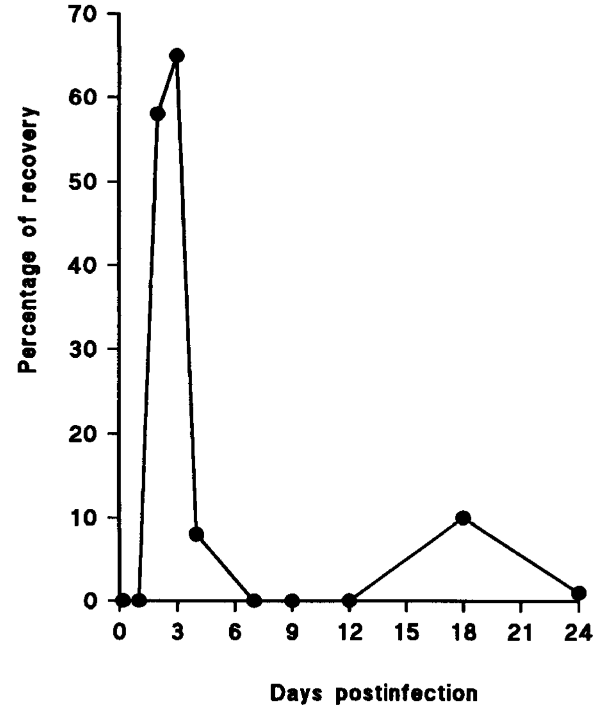

FIG. 2. Percentage of *T. canis* larvae recovered from liver of guinea-pigs studied at various times post-infection. Data obtained from five animals.

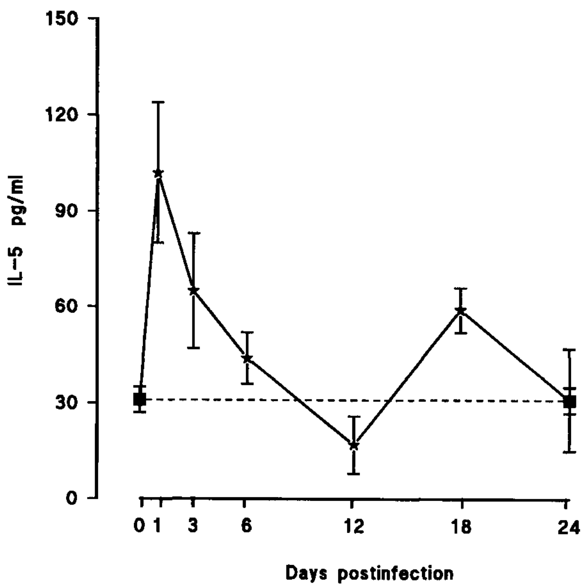

FIG. 3. IL-5 concentration in serum of *T. canis*-infected guinea-pigs ($n = 3-5$). Basal IL-5 concentrations were of 31 ± 4 pg/ml ($n = 12$).

drastically reduced, even when determined 18 days after infection (Table 1). No inhibition of eosinophil counts was observed when the animals were inoculated with the irrelevant antibody at the time of infection (Table 1).

Fig. 4 shows the comparative results of eosinophilia obtained when the antibody was given 3 days or 17 days after egg inoculation. The antibody given at 3 days after infection induced a high percentage of inhibition in eosinophil counts in all the compartments analysed 18 days after infection (Fig. 4A). However, when TRFK-5 was administered to the infected animals on day 17 post-infection (thus 1 day before sacrifice), a significant inhibition in number and percentage of eosinophils was observed only in the blood ($p = 0.030$) (Fig. 4B). A small non-significant decrease was seen in BALF ($p = 0.790$) and peritoneum ($p = 0.222$). Moreover, the number of mature eosinophils in bone marrow increased by 140% ($p = 0.038$).

As demonstrated in Fig. 4B, the behaviour of eosinophilia in BALF was completely different from that observed in blood. Thus, to better understand the eosinophilia in the lungs of infected animals, we monitored eosinophil numbers in BALF after administration of TRFK-5 at the same time, or 1, 3, 12 or 17 days after infection. The animals were sacrificed 18 days after infection. In another group, TRFK-5 was administered 18 days post-infection and the animals were sacrificed 6 days later. When the mAb was administered at the same time or 1 or 3 days post-infection there was a significant inhibition in the number of eosinophils (Fig. 5). These data show

**Table 1.** Eosinophils in *T. canis*-infected guinea-pigs treated or untreated with TRFK-5

| Compartment | Time of sacrifice (days) | Days of treatment with TRFK-5 after egg administration: Non-treated | 0 ($n$=6) | 1 ($n$=4) | 3 ($n$=4) | 12 ($n$=5) | 17 ($n$=5) | 18 ($n$=4) | Irrelevant Ab at the time of infection |
|---|---|---|---|---|---|---|---|---|---|
| Blood | 18 | 12.18 ± 5.28 | 0.14 ± 0.14* | 0.26 ± 0.26* | 0.17 ± 0.17* | 0.15 ± 0.15* | 2.59 ± 0.80* | - | 13.32 ± 4.54 |
| | 24 | 10.08 ± 2.85 | - | - | - | - | - | 0.15 ± 0.15* | |
| BALF | 18 | 16.50 ± 4.42 | 0.36 ± 0.10* | 0.79 ± 0.24* | 1.22 ± 0.50* | 7.07 ± 2.39 | 15.24 ± 4.36 | - | 29.31 ± 15.90 |
| | 24 | 6.57 ± 1.68 | - | - | - | - | - | 10.33 ± 4.10 | |
| Peritoneal cavity | 18 | 12.31 ± 2.35 | 1.47 ± 0.76* | 0.48 ± 0.35* | 0.46 ± 0.27* | 2.89 ± 0.94* | 10.42 ± 2.21 | - | 16.09 ± 4.00 |
| | 24 | 12.44 ± 2.33 | - | - | - | - | - | 3.79 ± 1.58* | |
| Bone marrow | 18 | 9 ± 1 | 3 ± 1* | 1.25 ± 0.25* | 2.6 ± 1.3* | 5 ± 1 | 15 ± 2 | - | 9 ± 1 |
| | 24 | 6 ± 1 | - | - | - | - | - | 2 ± 0.7* | - |

In blood BALF and peritoneal cavity the values represent mean ± S.E.M. × $10^5$ $ml^{-1}$ and in bone marrow mean ± S.E.M. of the percentage of mature eosinophils. *$p < 0.05$.

that the inhibition of the first peak of IL-5 which appeared at 1 to 3 days after infection as shown in Fig. 3, is also very important for the establishment of eosinophilia in the lungs. However, when the mAb was administered 12, 17 or 18 days after infection there was no significant inhibition in the numbers of eosinophils in BALF (Fig. 5), showing that once established, the eosi-

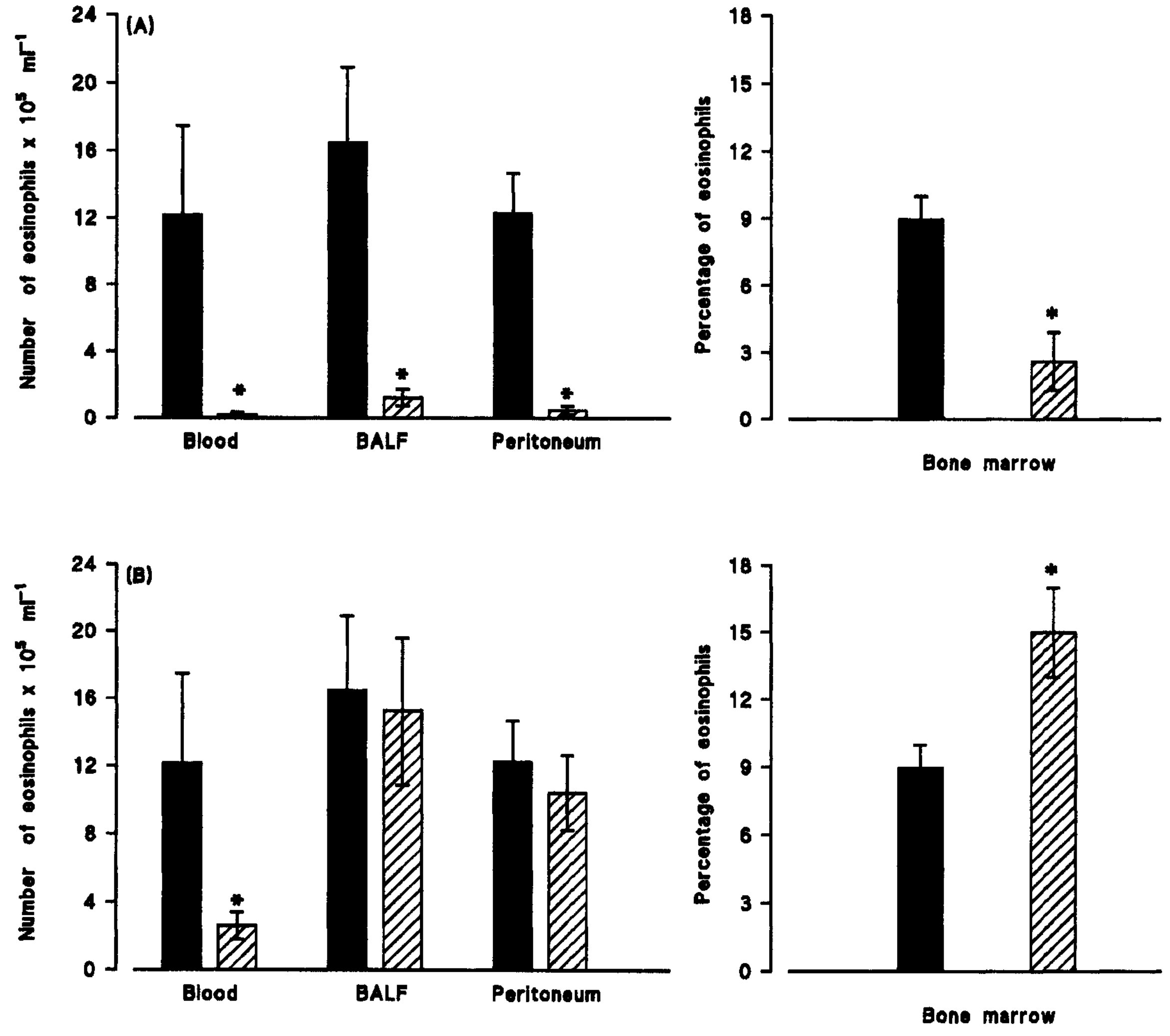

FIG. 4. Number of eosinophils in blood, BALF and peritoneum and percentage of eosinophils in bone marrow of *T. canis*-infected guinea-pigs submitted or not to treatment with TRFK-5. (A) 2 mg/animal at 3 days post-infection; (B) 2 mg/animal 17 days post-infection. The treated and control animals were sacrificed 18 days after infection. Asterisks indicate a significant difference from infected controls ($n$=5–6) and from animals treated with TRFK-5 ($n$=4–5). *$p < 0.05$ and **$p < 0.01$.

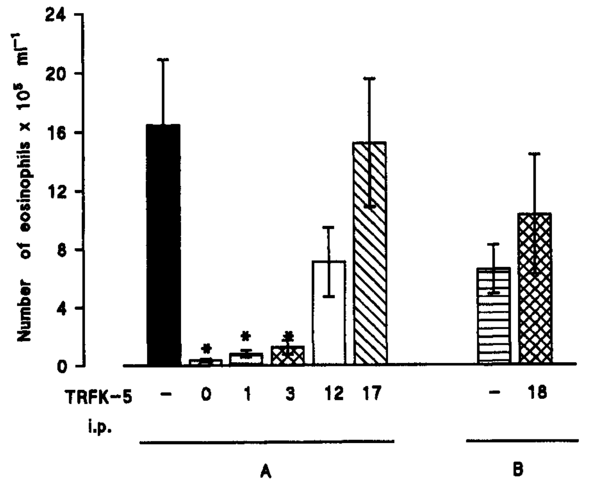

FIG. 5. Number of eosinophils in BALF of *T. canis*-infected guinea-pigs submitted or not to treatment with TRFK-5. (A) animals were sacrificed 18 days post-infection and (B) 24 days after infection. Asterisks indicate a significant difference from infected control and from animals treated with TRFK-5 ($p < 0.01$).

nophilia persists in lungs, probably by the secretion of IL-5 from cells localized in the lung microenvironment.

*Histopathological analysis:* The treatment of *T. canis*-infected animals with irrelevant antibody showed a widespread eosinophilic infiltration as in untreated animals (Fig. 6A,B). However, the treatment of animals with TRFK-5 at the same time of infection, or 1 day or 3 days later led to a complete inhibition of eosinophil infiltration in the lung parenchyma (Fig. 6C). By contrast, the mononuclear cell infiltration in the lungs was not modified. When the infected guinea-pigs received TRFK-5 1 day before sacrifice (or 17 days post-infection), eosinophil infiltration in the lung parenchyma was also inhibited (Fig. 6D) but not to the same extent as observed in the group receiving TRFK-5 given at the time of infection or 3 days later. Thus, the histological determination of eosinophil infiltration in these lungs corroborates a reduction but not a sizeable inhibition of eosinophil numbers as observed in the BALF of the same infected animals.

### Discussion

The results of the present study show that in our experimental model widespread eosinophilia follows the infection of guinea-pigs with second stage eggs from *T. canis*, as also noted in humans and in other experimental animals.[7,17] *T. canis* is a potent stimulus for systemic eosinophilia, since blood, BALF, peritoneum and all tissues examined (kidney, eyes, spleen, thymus, heart; data not shown) and muscle, as reported by other investigators,[18] were infiltrated.

The factors responsible for *in vivo* eosinophil accumulation at inflammatory sites have been poorly defined, although T lymphocytes and mast cells appear to be involved in eosinophilia.[19,20] IL-5, a T cell-derived factor that regulates B cell functions, is an eosinophil differentiation factor[11] as well as a stimulating and survival-prolonging factor specific for eosinophils *in vitro*.[12] Also, several investigators have demonstrated that systemic eosinophilia in mice infected with parasites is mediated by IL-5 produced in response to the infection.[21,22] In the present study, the i.p. administration of the TRFK-5 antibody markedly inhibited the widespread eosinophilia observed in *T. canis*-infected guinea-pigs, indicating that IL-5 participated in a guinea-pig model of VLMS eosinophilia.

Most of the *T. canis* larvae which penetrated the intestinal wall had migrated into the liver within 72 h after inoculation as demonstrated here and elsewhere.[23] It is apparently during this interval that the worm provides the signals to cytokine-producing cells, which in turn trigger increased serum levels of specific cytokine as demonstrated here for IL-5, 24 to 72 h after infection. The signals may be provided directly by the invading parasite or by cells in response to the parasite. The cytokine pattern that develops at this early stage, probably induced by a T-cell independent pathway, may also influence the pattern of T cell differentiation into a Th2 type, which may be responsible for the second peak of IL-5 observed in our experimental model (Fig. 3), although a second cycle of larval invasion (Fig. 2) with a rapid peak of IL-5 liberation cannot be ruled out.

Thus, our results suggest that the eosinophilia against helminth larvae may be initiated by the release of IL-5 when the parasites migrate from the intestine to the liver by stimulation of specific cell populations. Then, an early release of IL-5 quickly induces eosinophil recruitment, probably first from the stored mature eosinophil pool from vascular endothelium or by the mobilization of eosinophils from extravascular sites to the blood. This fact could explain why we found increased eosinophils first in blood and later in other compartments. The early IL-5 release may also serve as a signal for eosinophil differentiation and maturation in bone marrow. The time interval observed between the first peak of IL-5 release and the increase of eosinophils in blood coincides with that reported to be necessary for eosinophil differentiation and maturation *in vitro*.[12] Increased eosinophil production and liberation into blood and other tissues occurs

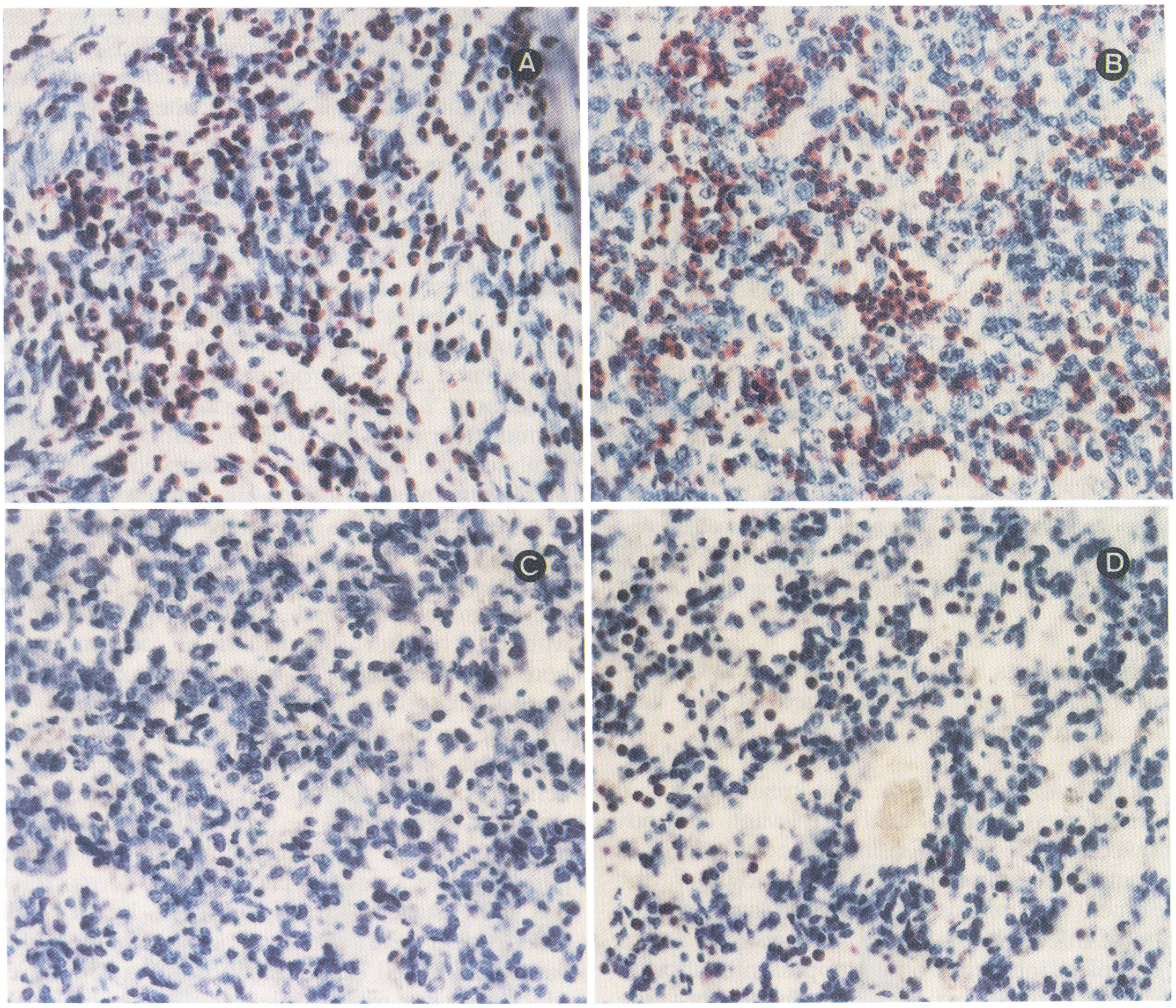

FIG. 6. (A) photomicrographs of lung parenchyma from guinea-pigs infected for 18 days with *T. canis*; (B) infected animals which were treated with irrelevant antibody at the time of infection; (C) infected animals which were treated with the mAb TRFK-5 at 3 days after infection; (D) mAb administration 17 days after infection. The animals were sacrificed 18 days after infection. Note the intense eosinophil infiltration into the lung in A and B, the inhibition of eosinophils in C and the reduction of eosinophils in D.

thereafter. Thus, early and later IL-5 release provides a necessary level of this cytokine, which is involved in the maintenance of eosinophilia. We may assume that the inhibition of the first peak of IL-5 release by TRFK-5 does not permit the subsequent T cell stimulation and differentiation. This may explain the long-lasting effect of TRFK-5 treatment observed here and also reported by others.[8] In agreement with our results, there is an important observation of Svetic *et al.*[24] showing that a specific and highly reproducible IL-5 gene expression pattern is detectable in Peyer's patches by 6 to 12 h after *Heligmosomoides polygyrus* infection. The early increase in IL-5 gene expression after infection was probably T cell-independent, inasmuch as it was observed in Peyer's patches of congenitally athymic mice and of conventional mice treated with anti-CD4 and anti-CD8 mAb. Moreover, Kusama *et al.*[25] have observed two peaks of eosinophilia in normal and athymic mice, and suggested that IL-5 observed in the first peak was produced by cells other than CD4 T cells, since anti-CD4 and anti-CD3 mAb reduced only the second peak of eosinophilia in normal mice and slightly reduced the first peak of eosinophilia in both normal and nu/nu mice. The local lung cells producing IL-5 may also help us to explain the reason why 12, 17 or 18 days post-infection TRFK-5 treatment only partially inhibits, or does not inhibit eosinophil infiltration into the lungs, as demonstrated in Figs 5 and 6. We may suggest that when eosinophils have already migrated to the lungs, TRFK-5 has no power to inhibit eosinophilia, which is also under control of local lung cells producing IL-5. In this way, only one later TRFK-

5 treatment may not be sufficient to modify the lung parenchyma microenvironment, since *T. canis* antigens have already stimulated some cell populations to produce IL-5, as demonstrated by Kusama *et al.*[25] These results suggest that eosinophilia in lungs is under the control of different factors when compared to that observed in blood and the peritoneal cavity.

One of the most important results obtained here was the inhibition of circulating eosinophil numbers by the different mAb treatments, even when the antibody was given at the peak of blood eosinophilia, which was accompanied by an increase of mature eosinophils in bone marrow. This suggests that IL-5, apart from being required for the terminal differentiation of eosinophils in bone marrow,[26] is also likely to drive eosinophils from the bone marrow to the blood and then to the tissues, probably by up-regulating VLA-4 expression in eosinophils. Moser *et al.*[16] have demonstrated that in order to acquire the ability to transmigrate, eosinophils must be primed with cytokines such as IL-5, IL-3 or GM–CSF for expression of adhesion molecules such as VLA-4. Recently, Pretolani *et al.*[27] have indeed shown that an anti-VLA-4 antibody suppresses eosinophil recruitment to lung in the guinea-pig and, as a consequence, inhibits the accompanying bronchopulmonary hyperresponsiveness.

### References

1. Nutman TB, Ottesen EA, Cohen SG. The eosinophil, eosinophilia, and eosinophil-related disorders. *Allergy Proc* 1989; **10:** 47–62.
2. Glickman LT, Schantz PM. Epidemiology and pathogenesis of zoonotic toxocariasis. *Epidem Rev* 1981; **3:** 230–250.
3. Beaver P, Snyder H, Carrera G, Dent J, Lafferty J. Chronic eosinophilia due to visceral larva migrans. *Pediatrics* 1952; **9:** 7–19.
4. Limaye AP, Abrams JS, Silver JE, Ottesen EA, Nutman TB. Regulation of parasite-induced eosinophilia: selectively increased interleukin 5 production in helminth-infected patients. *J Exp Med* 1990; **172:** 399–402.
5. Steel C, Nutman TB. Regulation of IL-5 in onchocerciasis: a critical role for IL-2. *J Immunol* 1993; **150:** 5511–5518.
6. Yamaguchi Y, Matsui T, Kasahara T, *et al. In vivo* changes of hemapoietic progenitors and the expression of the interleukin 5 gene in eosinophilic mice infected with *Toxocara canis. Exp Hematol* 1990; **18:** 1152–1157.
7. Parsons JC, Coffman RL, Grieve RB. Antibody to interleukin 5 prevents blood and tissue eosinophilia but not liver trapping in murine larval toxocariasis. *Parasite Immunol* 1993; **15:** 501–508.
8. Coffman RL, Seymour BWP, Hudak S, Jackson J, Rennick D. Antibody to interleukin-5 inhibits helminth-induced eosinophilia in mice. *Science* 1989; **245:** 308–310.
9. Sher A, Coffman RL, Hieny S, Cheever AW. Ablation of eosinophil and IgE responses with anti-IL-5 or anti-IL-4 antibodies fails to affect immunity against *Schistosoma mansoni* in the mouse. *J Immunol* 1990; **145:** 3911–3916.
10. Urban Jr JF, Katona IM, Paul WE, Finkelman FD. Interleukin 4 is important in protective immunity to a gastrointestinal nematode infection in mice. *Proc Natl Acad Sci USA* 1991; **88:** 5513–5517.
11. Yamaguchi A, Suda T, Suda J, *et al.* Purified interleukin 5 (IL-5) supports the terminal differentiation and proliferation of murine eosinophilic precursors. *J Exp Med* 1988; **167:** 43–56.
12. Yamaguchi Y, Hayashi Y, Sugama Y, *et al.* Highly purified murine interleukin 5 (IL-5) stimulates eosinophil function and prolongs *in vitro* survival. IL-5 as an eosinophil chemotactic factor. *J Exp Med* 1988; **167:** 1737–1752.
13. Rothenberg ME, Petersen J, Stevens RL, Silberstein DS, McKenzie DT, Austen KF, Owen WF. IL-5-dependent conversion of normodense human eosinophils to the hypodense phenotype uses 3T3 fibroblasts for enhanced viability, accelerated hypodensity, and sustained antibody-dependent cytotoxicity. *J Immunol* 1989; **143:** 2311–2316.
14. Collins PD, Weg VB, Faccioli LH, Watson ML, Moqbel R, Williams TJ. Eosinophil accumulation induced by human interleukin-8 in the guinea pig *in vivo. Immunology* 1993; **79:** 312–318.
15. Sehmi R, Wardlavo AJ, Cromwell O, Kurihara K, Waltmann P, Kay AB. Interleukin-5 selectively enhances the chemotactic response of eosinophils obtained from normal but not eosinophilic subjects. *Blood* 1992; **79:** 2952–2959.
16. Moser R, Fehr J, Bruijnzeel PLB. IL-4 controls the selective endothelium-driven transmigration of eosinophils from allergic individuals. *J Immunol* 1992; **149:** 1432–1438.
17. Olson LJ, Schulz CW. Nematode induced hypersensitivity reactions in guinea pigs: onset of eosinophilia and positive Schultz–Dale reactions following graded infection with *Toxocara canis. Ann N Y Acad Sci* 1963; **113:** 440–455.
18. Kayes SG, Oaks JA. Development of the granulomatous response in murine toxocariasis. I. Initial events. *Am J Pathol* 1978; **93:** 277–294.
19. Basten A, Beeson PB. Mechanisms of eosinophilia. II. Role of the lymphocyte. *J Exp Med* 1970; **131:** 1288–1305.
20. Plaut M, Pierce JH, Watson CJ, Hanley-Hyde J, Nordan RP, Paul WE. Mast cell lines produce lymphokines in response to cross-linkage of Fc epsilon RI or to calcium ionophoras. *Nature* 1989; **339:** 64–67.
21. Sher A, Coffman RL, Hieny S, Scott P, Cheever AW. Interleukin 5 is required for the blood and tissue eosinophilia but not granuloma formation induced by infection with *Schistosoma mansoni. Proc Natl Acad Sci USA* 1990; **87:** 61–65.
22. Herndon FJ, Kayes SG. Depletion of eosinophils by anti-IL-5 monoclonal antibody treatment of mice infected with *Trichinella spiralis* does not alter parasite burden or immunologic resistance to reinfection. *J Immunol* 1992; **149:** 3642–3647.
23. Oshima T. Standardization of techniques for infecting mice with *Toxocara canis* and observations on the normal migration routes of the larvae. *J Parasitol* 1961; **47:** 652.
24. Svetic A, Madden KB, Zhou XD, *et al.* A primary intestinal helminthic infection rapidly induces a gut-associated elevation of Th2-associated cytokines and IL-3. *J Immunol* 1993; **150:** 3434–3441.
25. Kusama Y, Takamoto M, Kasahara T, Takatsu K, Nariuchi H, Sugane K. Mechanisms of eosinophilia in BALB/c-nu/+ and congenitally athymic BALB/c-nu/nu mice infected with *Toxocara canis. Immunology* 1995; **84:** 461–468.
26. Rennick DM, Thompson-Snipes L, Coffman RL, Seymour BWP, Jackson JD, Hudak S. *In vivo* administration of antibody to interleukin-5 inhibits increased generation of eosinophils and their progenitors in bone marrow of parasitized mice. *Blood* 1990; **76:** 312–316.
27. Petrolani M, Ruffié C, Lapa e Silva JR, Joseph D, Lobb RR, Boris Vargaftig B. Antibody to very late activation antigen 4 prevents antigen-induced bronchial hyperreactivity and cellular infiltration in guinea pig airways. *J Exp Med* 1994; **180:** 795–805.

ACKNOWLEDGEMENTS. This work was funded by grant 300652/85-2 from Conselho Nacional de Desenvolvimento Científico e Tecnológico (CNPq) and Grant 92/5105-7 from Fundação de Amparo à Pesquisa do Estado de São Paulo (FAPESP). We wish to thank Mrs M.A. Fernandes for technical assistance, Mrs M.M.O. Rossi for the histological sections, M. Costa Gonçalves for the photography artwork, and Dr P. Minoprio, Institut Pasteur, for kindly providing TRFK-5.

**Received 12 October 1995; accepted 17 November 1995**
